# Effect of ertugliflozin on renal function and cardiovascular outcomes in patients with type 2 diabetes mellitus: A systematic review and meta-analysis

**DOI:** 10.1097/MD.0000000000033198

**Published:** 2023-03-10

**Authors:** Qian Cheng, Shupeng Zou, Chengyang Feng, Chan Xu, Yazheng Zhao, Xuan Shi, Minghui Sun

**Affiliations:** a Department of Pharmacy, Tongji Hospital, Tongji Medical College, Huazhong University of Science and Technology, Wuhan, China.

**Keywords:** cardiovascular event, eGFR, meta-analysis, safety, sodium-glucose cotransporter type-2 (SGLT2) inhibitors, T2DM

## Abstract

The global prevalence of type 2 diabetes mellitus (T2DM) is growing yearly. The efficacy of ertugliflozin (ERT), a recently licensed anti-diabetic drug, has been widely reported. However, additional evidence-based data is required to ensure its safety. In particular, convincing evidence on the effects of ERT on renal function and cardiovascular outcomes is needed.

**Methods:**

We searched PubMed, Cochrane Library, Embase, and Web of Science for randomized placebo-controlled trials of ERT for T2DM published up to August 11, 2022. Cardiovascular events here mainly refer to acute myocardial infarction and angina pectoris (AP) (including stable AP and unstable AP). The estimated glomerular filtration rate (eGFR) was used to measure renal function. The pooled results are risk ratios (RRs) and 95% confidence intervals (CIs). Two participants worked independently to extract data.

**Results:**

We searched 1516 documents and filtered the titles, abstracts, and full text, 45 papers were left. Seven trials met the inclusion criteria and were ultimately included in the meta-analysis. The meta-analysis found that ERT reduced eGFR by 0.60 mL·min^−1^·1.733 m^−2^ (95% CI: −1.02–−0.17, *P* = .006) in patients with T2DM when used for no more than 52 weeks and these differences were statistically significant. Compared with placebo, ERT did not increase the risk of acute myocardial infarction (RR 1.00, 95% CI: 0.83–1.20, *P* = .333) and AP (RR 0.85, 95% CI: 0.69–1.05, *P* = .497). However, the fact that these differences were not statistically significant.

**Conclusion:**

This meta-analysis shows that ERT reduces eGFR over time in people with T2DM but is safe in the incidence of specific cardiovascular events.

## 1. Introduction

Diabetes is becoming more common worldwide, and the International Diabetes Federation predicts that 537 million people will have diabetes by 2021 (both diagnosed and undiagnosed). This figure is expected to rise by 46% to 783 million by 2045.^[1]^ Type 2 diabetes is becoming increasingly common across the world. Type 2 diabetes mellitus (T2DM) accounts for about 90% of the overall population.^[2,3]^

There are several complications associated with type 2 diabetes, which may be classified into 2 groups: microvascular and macrovascular. Cardiovascular disease is a macrovascular problem, whereas nephropathy is a microvascular consequence.^[4]^ According to current research, most T2DM patients die from cardiovascular disease, with coronary atherosclerotic heart disease and heart failure (HF) being the primary causes of mortality.^[5–8]^ Furthermore, recent research has indicated that people with diabetes are more likely than non-diabetics to experience HF and lower ejection fraction.^[9,10]^ Furthermore, a survey found that individuals with atherothrombosis and T2DM had a 30% greater risk of HF hospitalization than those with atherothrombosis but no T2DM.^[10]^ Also, diabetes is the primary cause of end-stage renal disease,^[11,12]^ and diabetes can result in chronic kidney disease (diabetic nephropathy), which involves 20 to 40% of diabetics.^[13,14]^ Interestingly, chronic kidney disease usually appears approximately ten years after type 1 diabetes has been diagnosed, but at that time, type 2 diabetes has already been diagnosed.^[5]^ Therefore, in the absence of overt symptoms, diabetic nephropathy has a decreased estimated glomerular filtration rate (eGFR) as one of its clinical diagnostic criteria. Similarly, when glycated hemoglobin (HbA1c) was >7.0%, each 1% rise in HbA1c was linked with a 38% increase in mortality risk from macrovascular events. When HbA1c was >6.5%, the risk of microvascular death increased by 40% for every 1% increase in HbA1c.^[15]^ As a result, renal function and cardiovascular events significantly influence the survival of type 2 diabetes patients.

Ertugliflozin (ERT) is a new generation of sodium-glucose cotransporter-2 inhibitors that can lower blood glucose by reducing glucose reabsorption in the kidney and increasing glucose excretion in the urine.^[16,17]^ Although the hypoglycemic impact of ERT has been extensively reported in previous research, there is no consensus on its effect on eGFR in T2DM patients. Linong Ji^[18]^ and Samuel Dagogo-Jack^[19]^ discovered that ERT when compared to a placebo group, slowed the drop in eGFR. However, in a VERTIS CV sub-study,^[20]^ eGFR fell first in the ERT group compared to the placebo group and remained steady above baseline levels throughout the therapy. In the most recent meta-analysis of ERT, safety and tolerability have been reported.^[16]^ However, its safety studies did not include its effect on eGFR. So, what effect does ERT have on eGFR in T2DM patients? We conduct this meta-analysis to address that question. In addition, issues about the influence on cardiovascular events in T2DM patients must be investigated. According to the findings of the VERTIS CV Trial, ERT reduced the total occurrence rate of hospitalization for heart failure (HHF) (risk ratio [RR] 0.70, 95% confidence interval [CI]: 0.56–0.87) and total HHF/CV mortality (RR 0.83, 95% CI: 0.72–0.96).^[6]^ However, the effect on other cardiovascular events, such as acute myocardial infarction (AMI) and angina pectoris (AP), has not been observed. This meta-analysis will also address these concerns.

## 2. Patients and methods

We performed a systematic review and meta-analysis according to the preferred reporting items for systematic review and meta-analyses guidelines. The systematic review protocol has been registered in the PROSPERO database (International Prospective Register of Systematic Reviews, https://www.crd.york.ac.uk/prospero; registration number CRD42022332437).

## 3. Data sources and searches

From conception through August 2022, we did a comprehensive search of the PubMed, Cochrane Library, Embase, and Web of Science databases using the terms “ertugliflozin,” “type 2 diabetes,” and associated phrases. We manually reviewed the references of chosen research, relevant meta-analyses, and review papers, to verify that no eligible trials were ignored. The search is restricted to English language articles only. EndNote X9 (Clarivate, Philadelphia, PA) is used to manage and edit pertinent documents, as well as to eliminate duplicate files.

## 4. Study inclusion and exclusion criteria

The following were the criteria for inclusion in the literature. T2DM patients are the target recipient; the individuals were 18 years old and had HbA1c levels ranging from 8.0 to 8.21%; the papers included were randomized controlled trials that compared ERT to placebo; and renal markers of eGFR or cardiovascular events (AMI and AP) have been documented. The following were the exclusion criteria: all participants had type 1 diabetes mellitus; the research was not a randomized controlled experiment; and reusing raw data or doing secondary analysis.

## 5. Data extraction and quality assessment

Two reviewers did the initial screening of the literature by reading the titles and abstracts of the literature back-to-back. Documents with incomplete information in the title and abstract, as well as those that met the initial screening criteria, were then read in detail. Finally, for literature that met the inclusion criteria, its authors, year of publication, clinical trial registration number, interventions, controls, mean age, percentage of men, mean glycated hemoglobin, mean body mass index (BMI), mean eGFR, and length of treatment were recorded. The final renal function index was eGFR; the eligible cardiovascular outcome events were AMI and AP (including stable AP and unstable AP).

The Cochrane Collaboration’s risk of bias tool was used to evaluate the included literature regarding random sequence generation, allocation concealment, blinding quality (including for participants, investigators, and analysts), data integrity, selective reporting, and other biases. Each article scored high, low, or uncertain based on the related criteria. Two reviewers worked separately on literature screening and data extraction, and discrepancies were addressed through conversation. If the discussion failed to achieve a consensus, a third reviewer was consulted. R4.2.1 was used for the analysis (https://www.r-project.org/).

## 6. Data synthesis and analysis

Continuous variables were expressed as mean ± standard deviation (SD). When only standard errors were reported, the following formula was used for conversion: SD = SE·√n. weighted mean differences (WMDs) and associated 95% CI were selected as the pooled effect size for continuous variables. For dichotomous variables, RR and their 95% CI were used to describe the risk of cardiovascular events. Stata SE17.0 (Stata Corporation, College Station, TX) was used for data analysis, and *P* < .05 was considered a statistically significant difference.

Heterogeneity was calculated using the Cochrane *Q* statistic and expressed as *I*^2^. When *I*^2^ ≤ 40%, it was considered low heterogeneity; when 40% < *I*^2^ ≤ 70%, it was regarded as moderate heterogeneity; when 70% < *I*^2^ ≤ 100%, it was considered as high heterogeneity. Suppose the *P* value was ≥ .05 and *I*^2^ ≤ 40%, it indicated less heterogeneity among the included trials, and a fixed-effects model was used; if not, we used a random-effects model. When there is heterogeneity, we deal with it through these 2 approaches. Subgroup analysis was done based on age (≥55 and <60 years, ≥60 years), duration of T2DM (≥7 years, <7 years), dose (ERT 5 mg, ERT 15 mg), BMI (≥31, <31), HbA1c (≥8.1% and < 8.5%, <8.1%) and eGFR (≥30 and <60, ≥60) (see Figures S1–S7, Supplemental Digital Content, http://links.lww.com/MD/I627; http://links.lww.com/MD/I628; http://links.lww.com/MD/I629; http://links.lww.com/MD/I630; http://links.lww.com/MD/I631; http://links.lww.com/MD/I632; http://links.lww.com/MD/I633, which showed the results of subgroup analysis). Sensitivity analysis was conducted to find the source of heterogeneity by eliminating the included literature one by one (see Figure S8, Supplemental Digital Content, http://links.lww.com/MD/I634, which showed the sensitivity analysis result). However, all subgroup and interaction analyses (*P* for interaction) in this meta-analysis were done to obtain more credible results.

The Egger test evaluated publication bias in Stata SE17.0. *P* < .05 indicates possible publication bias (see Figure S9, Supplemental Digital Content, http://links.lww.com/MD/I635, which showed the result of the Egger test).

## 7. Results

### 7.1. Characteristics of the included literature

**Figure 1.**
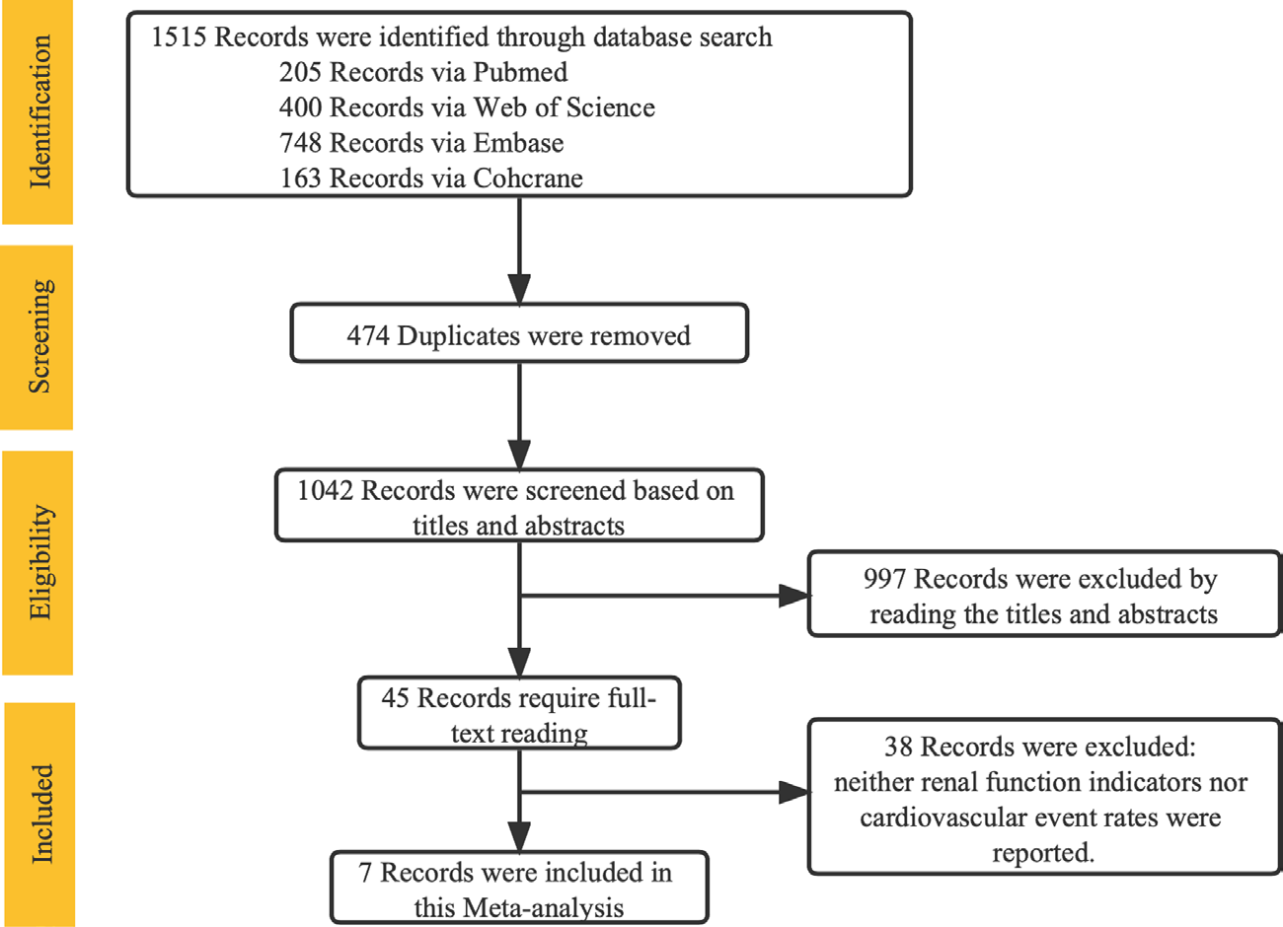
PRISMA flow diagram of eligible randomized controlled trials. PRISMA = Preferred Reporting Items for Systematic Review and Meta-Analyses.

We searched for a total of 1516 papers. After removing 474 duplicates, 1042 remained. The titles and abstracts of these 1042 papers were screened, and 45 papers were retained for further review. Finally, after a detailed reading of these 45 papers, 7 met the inclusion criteria (Fig. 1).

The characteristics of the included trials are presented in Table 1. Finally, 7 RCTs were included in this meta-analysis, with a total of 11,091 T2DM patients.^[16,18,21–25]^ Eleven thousand ninety-one patients with T2DM were randomly assigned to receive 5 mg of ERT, 15 mg of ERT, or a placebo. The mean age of the participants ranged from 54 to 68. Four RCTs included participants mainly from North America, South America, Europe, and South Africa, 1 RCT had subjects mainly from Asia (including mainland China, Hong Kong, Taiwan, South Korea, and the Philippines), and the other 2 RCTs did not give relevant information. The 7 RCTs’ glycated hemoglobin baseline averages varied from 8.0 to 8.21%. Six RCTs had baseline mean eGFR values between 46.6 and 99.3 mL·min^−1^·1.73 m^−2^, and no information was reported for the other RCT. All literature was published between 2015 and 2021. Table 1 provides more details for the remainder.

**Table 1:** Characteristics of included randomized controlled trials.

Study	Year	NCT number	N	Intervention	Control	Age (mean ± SD)	Men (n)	HbA1C (mean ± SD) (%)	BMI (mean ± SD) (kg·m^−2^)	eGFR (mean ± SD) (mL·min^−1^·1.73 m^−2^)	Treatment duration (wk)
Amin et al	2015	NCT01059825	328	ERT – 5mg	Placebo	54.44 ± 8.63	213 (64.9%)	8.11 ± 1.14	30.4 ± 5.41	/	12
Steven et al	2017	NCT01958671	461	ERT – 5mg ERT – 15mg	Placebo	56.4 ± 11.0	261 (56.6%)	8.21 ± 0.98	33.0 ± 6.7	87.7 ± 18.6	26
Samuel et al	2017	NCT02036515	462	ERT – 5mg ERT – 15mg	Placebo	59.1 ± 9.0	263 (56.9%)	8.0 ± 0.9	30.8 ± 6.0	87.9 ± 16.9	52
George et al	2018	NCT01986855	467	ERT – 5mg ERT – 15mg	Placebo	67.3 ± 8.6	231 (49.5%)	8.2 ± 0.9	32.5 ± 6.1	46.6 ± 8.8	52
Silvina et al	2019	NCT02033889	621	ERT – 5mg ERT – 15mg	Placebo	56.6 ± 8.8	288 (46.4%)	8.12 ± 0.90	31.1 ± 4.7	90.5 ± 19.3	26
Linong et al	2019	NCT02630706	506	ERT – 5mg ERT – 15mg	Placebo	56.5 ± 9.1	281 (55.5%)	8.1 ± 0.9	26.0 ± 3.2	99.3 ± 19.7	26
David et al	2021	NCT01986881	8246	ERT – 5mg ERT – 15mg	Placebo	64.4 ± 8.1	5769 (69.7%)	8.2 ± 1.0	32.0 ± 5.4	76.0 ± 20.9	52

BMI = body mass index, eGFR = estimated glomerular filtration rate, ERT = ertugliflozin, SD = standard deviation.

### 7.2. Quality assessment of individual trials

**Figure 2.**
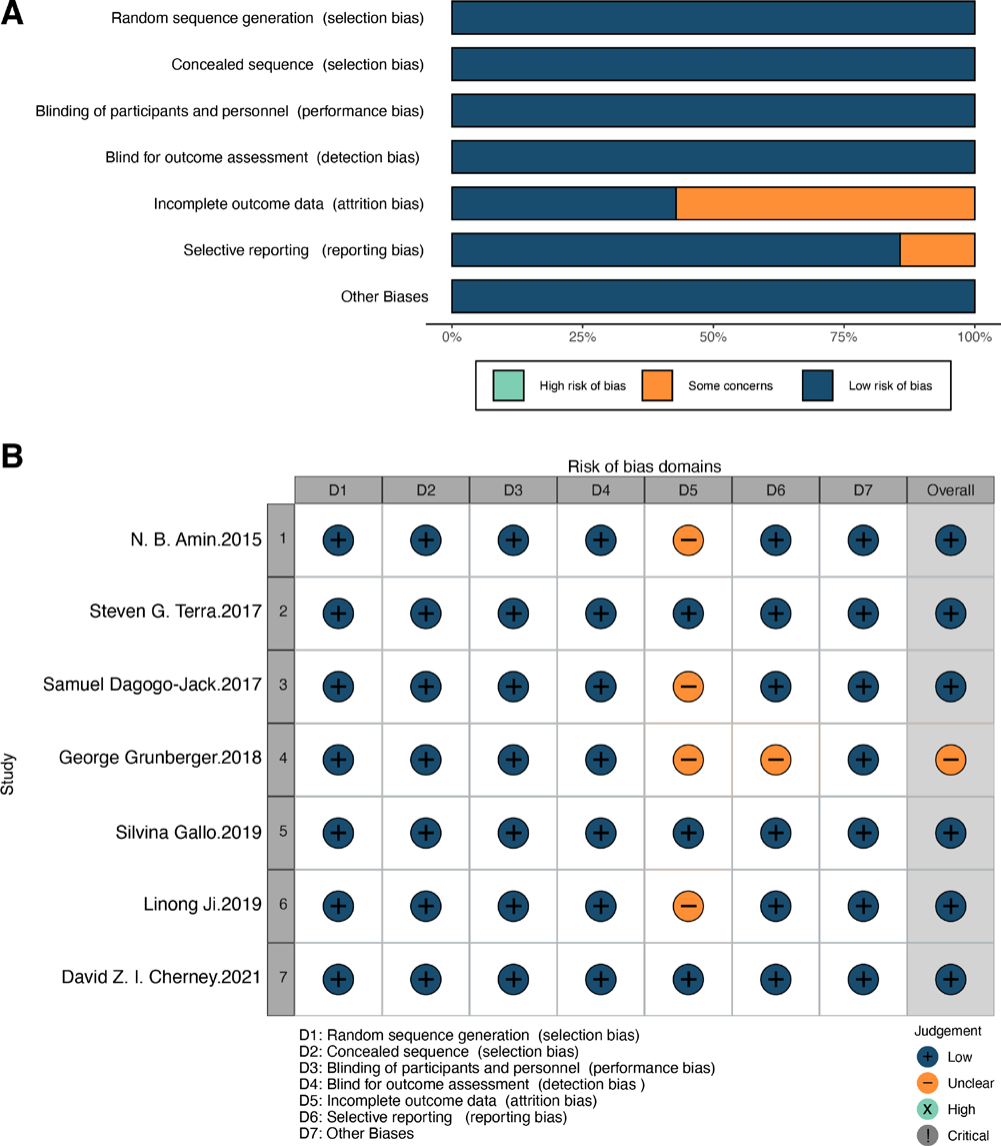
(A) Risk of bias graph and (B) risk of bias summary for included RCTs. RCT = randomized controlled trial.

The Cochrane Collaboration tool was used to evaluate the included studies, and Figure 2A and B summarize the risk of bias. All the examined literature had low chance of biases related to random sequence generation, allocation concealment, blinded implementation quality, and other biases. Regarding the completeness of the data, 3 RCTs were low-risk, and the remaining 4 did not provide sufficient information to be judged. For reporting bias, only 1 RCT was unclear, and the remaining 5 were low-risk.

### 7.3. Effect of ERT on laboratory changes in renal function

**Figure 3.**
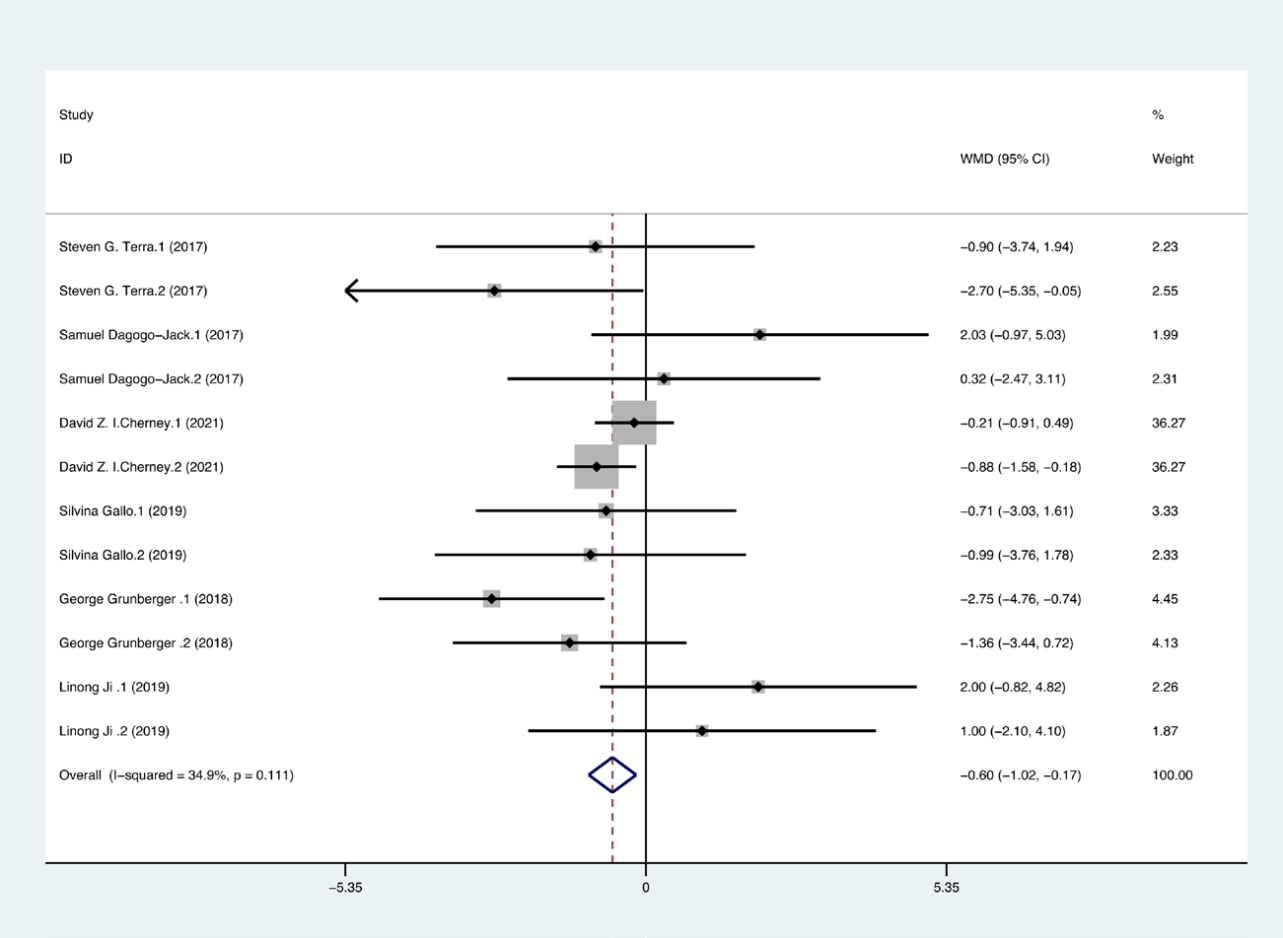
Forest plot of effects of ertugliflozin on eGFR in patients with T2DM. eGFR = estimated glomerular filtration rate, T2DM = type 2 diabetes mellitus.

For treatment durations up to 52 weeks, the analysis revealed a significant reduction in eGFR in the ERT group compared to the placebo group (SMD −0.60, 95% CI: −1.02–−0.17, *P* = .006) (Fig. 3). Subsequently, we performed subgroup analyses for age (see Figure S1, Supplemental Digital Content, http://links.lww.com/MD/I627), duration of T2DM (see Figure S2, Supplemental Digital Content, http://links.lww.com/MD/I628), dose (see Figure S3, Supplemental Digital Content, http://links.lww.com/MD/I629), BMI (see Figure S4, Supplemental Digital Content, http://links.lww.com/MD/I630), glycated hemoglobin (see Figure S5, Supplemental Digital Content, http://links.lww.com/MD/I631), eGFR (see Figure S6, Supplemental Digital Content, http://links.lww.com/MD/I632), and length of treatment (see Figure S7, Supplemental Digital Content, http://links.lww.com/MD/I633), respectively. Subgroup analysis showed that in terms of eGFR, there was a more significant decline in those ≥60 years old (WMD −0.71, 95% CI: −1.18–−0.24, *P* = .003) than in those ≥55 and <60 years old (WMD −0.13, 95% CI: −1.10–0.85, *P* = .798); those with ≥7 years of T2DM duration (WMD −0.53, 95% CI: −0.97–−0.10, *P* = .016) than those with <7 years (WMD −1.86, 95% CI: −3.80–0.08, *P* = .060); those on ERT 15 mg (WMD −0.89, 95% CI: −1.49–−0.29, *P* = .004) than those on 5 mg (WMD −0.31, 95% CI: −0.91–0.29, *P* = .307). We also found that eGFR decreased more in those with BMI ≥ 31 kg·m^−2^ (WMD −0.77, 95% CI: −1.22–−0.33, *P* = .001) than in those <31 kg·m^−2^ (WMD 1.32, 95% CI: −0.13–2.78, *P* = .075); glycated hemoglobin ≥ 8.1% and <8.5% (WMD −0.62, 95% CI: −1.05–−0.18, *P* = .006) than those <8.1% (WMD −0.29, 95% CI: −2.07–1.49, *P* = .751); those with baseline eGFR ≥ 30 and <60 mL·min^−1^·1.73 m^−2^ (WMD −1.58, 95% CI: −2.70–−0.46, *P* = .006) than those ≥60 mL·min^−1^·1.73 m^−2^ (WMD −0.43, 95% CI: −0.89–0.02, *P* = .063); and more in those treated for 52 weeks (WMD −0.62, 95% CI: −1.07–−0.16, *P* = .008) than in those treated for 26 weeks (WMD −0.49, 95% CI: −1.60–−0.62, *P* = .384). ERT significantly reduced eGFR for BMI ≥ 31 kg·m^−2^ compared with BMI < 31 kg·m^−2^, with a *P* interaction of 0.007.

### 7.4. Effect of ERT on cardiovascular outcomes

**Figure 4.**
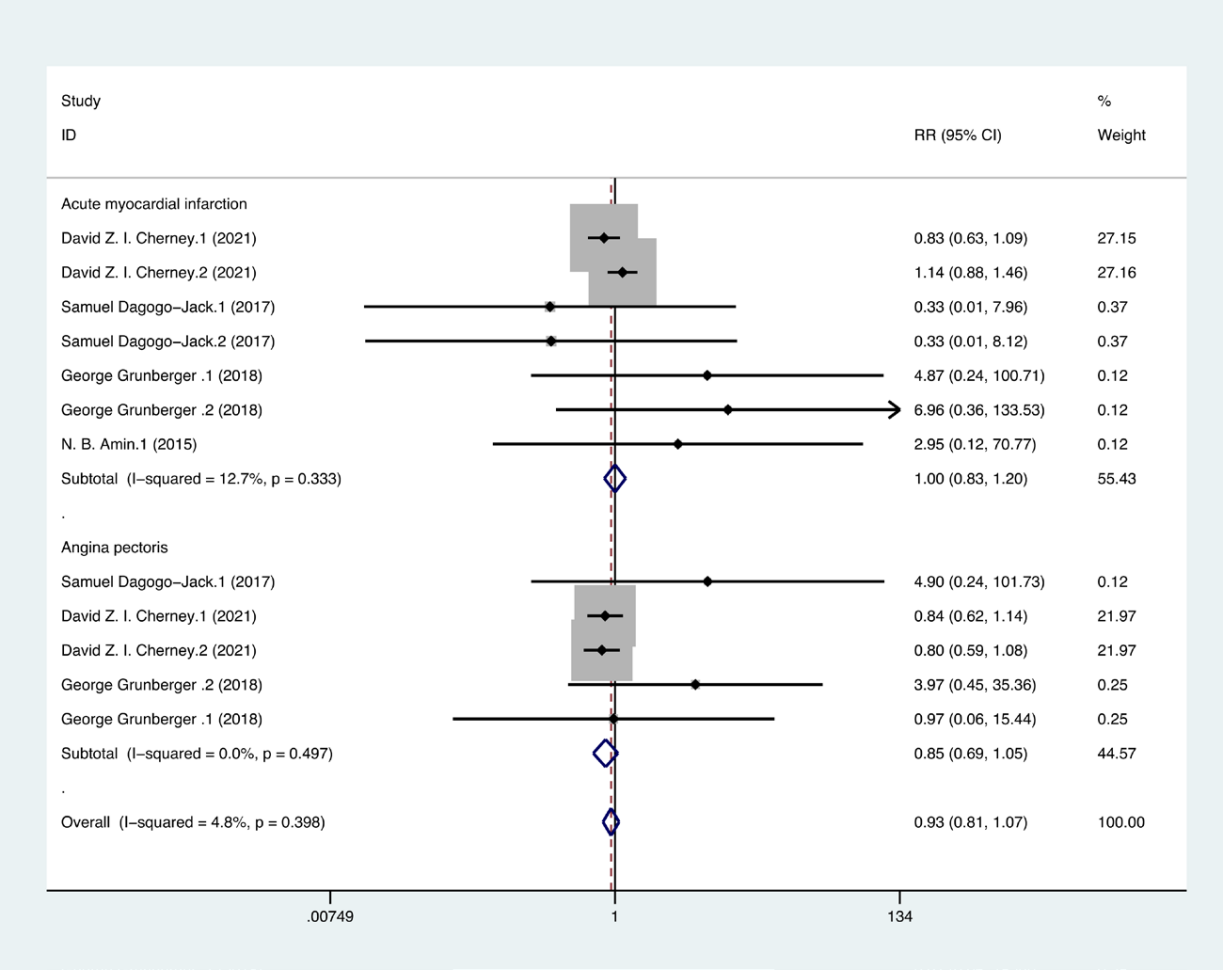
Forest plot of effects of ertugliflozin on the composite of AMI and AP of the T2DM participant. AMI = acute myocardial infarction, AP = angina pectoris, T2DM = type 2 diabetes mellitus.

A total of 9503 patients were included in the 4 RCTs which reported associated cardiovascular events. AMI and AP are the main cardiovascular events reported in the 4 RCTs. The pooled analysis did not show significant changes in the following cardiovascular events associated with ERT compared with placebo. However, the risk of AP (RR 0.85, 95% CI: 0.69–1.05, *P* = .497) showed a downward trend. There was no significant difference between ERT and placebo in the risk of AMI (RR 1.00, 95% CI: 0.83–1.20, *P* = .333) (Fig. 4).

## 8. Discussion

T2DM has produced a considerable disease burden by raising the risk of cardiovascular, renal, and other consequences; it has become the most severe public health concern in China and throughout the world, and it is one of the most severe chronic illnesses endangering human health today.^[26]^ According to published research, sodium-glucose cotransporter type-2 inhibitor (SGLT2i) is cardioprotective, and this protection is not dependent on its glucose-lowering actions.^[27]^ There is also some indication that SGLT2i can protect the kidneys of T2DM patients. A meta-analysis found that SGLT2is lower the risk of proteinuria, acute kidney damage, and renal transplantation in T2DM patients.^[28]^

ERT, a new generation of SGLT2i, has been proven to lower the risk of cardiovascular disease, particularly HHF hospitalization.^[29]^ However, the effect on AMI and AP (including stable AP and unstable AP) has not been documented. Furthermore, ERT has been shown to impact eGFR in T2DM patients. The findings of the VERTIS MONO Phase A study revealed that after 26 weeks of therapy, the mean (SD) eGFR change from baseline was 0.5 (11.8) and −1.3 (10.1) mL·min^−1^·1.73 m^−2^ for ERT 5 and 15 mg, respectively, compared to a change of 1.4 (11.2) mL·min^−1^·1.73 m^−2^ for placebo.^[22]^ VERTIS SITA2 showed similar findings.^[19]^ Another result from the VERTIS CV trial showed that after 52 weeks of treatment, the mean (SD) eGFR change from baseline was −0.5 (13.0) and −1.2 (13.0) mL·min^−1^·1.73 m^−2^ for ERT 5 and 15 mg, respectively compared with a change of −0.3 (13.2) mL·min^−1^·1.73 m^−2^ with placebo.^[20]^ As a result, the findings of previous studies on how ERT affects eGFR in T2DM patients are not entirely consistent. Therefore, this meta-analysis examined the available data to determine the relationship between ERT and associated cardiovascular events. In light of this, this meta-analysis also assessed the changes in eGFR and the risk of cardiovascular events in T2DM patients receiving ERT.

According to our findings, there was no apparent difference between the effects of ERT and placebo on AMI and AP (including stable AP and unstable AP) in people with type 2 diabetes. Especially in AP, ERT tends to reduce the risk of occurrence. The overall findings indicate that ERT decreases the risk of these 2 cardiovascular events (RR 0.93, 95% CI: 0.81–1.07, *P* = .398); however, this reduction is not statistically significant. Another meta-analysis revealed similar findings. Li Liu’s study concluded that ERT reduced systolic blood pressure by 2.57 mm Hg and diastolic blood pressure by 1.15 mm Hg in the T2DM population compared to the control group.^[30]^ According to the findings of the HONEST research, those with high blood pressure and diabetes are 2.8 times more likely to suffer cardiovascular disease than those who only have diabetes.^[31]^ These findings suggest that lowering blood pressure may decrease the risk of cardiovascular events in T2DM patients.^[32]^

Our results showed that ERT significantly reduced eGFR in patients with T2DM (SMD −0.60, 95% CI: −1.02–0.17, *P* = .006). This result was more reliable in T2DM patients with a BMI over 31 (*P*_interaction_ = 0.007). In addition, people with T2DM and one of the following conditions also showed a significant decrease in eGFR after treatment with ERT: ≥60 years old, those with ≥7 years of T2DM duration, those on ERT 15mg, glycated hemoglobin ≥ 8.1% and <8.5%, those with baseline eGFR ≥ 30 and <60 mL·min^−1^·1.73 m^−2^, those treated for 52 weeks (All *P* values < 0.05). The mechanism may be that ERT inhibits the reabsorption of glucose by the renal tubules, which in turn leads to a decrease in the eGFR. In the long term, these effects of ERT may help to protect the metabolic function of renal tubular cells and delay the decline of eGFR.^[33]^ These results suggest that those patients with T2DM who are overly obese, elderly, have had T2DM for a long time, have high baseline glucose levels, or have poor baseline renal function are more likely to experience a decrease in eGFR during treatment with ERT.

Our study had certain limitations, although being quite comprehensive, and the subgroup and sensitivity analysis results were consistent with the main pooled results. Because of a lack of data from the original trial, therapy beyond 52 weeks could not be examined. The results are somewhat restricted because there was no comparison of ERT to other SGLT2is. As a result, more convincing results require more extended treatment duration data and comparisons with other SGLT2is.

## 9. Conclusion

Our meta-analysis demonstrated that ERT was safe for AMI and AP. However, in terms of renal function, ERT produces a decline in eGFR over time, particularly in patients who are obese, old, have high basal glucose, and have a low baseline eGFR. As a result, while using ERT in these patients, greater attention should be paid to monitoring their renal function indicators to avoid renal damage.

## Author contributions

**Data curation:** Chengyang Feng.

**Investigation:** Chan Xu, Xuan Shi.

**Resources:** Yazheng Zhao.

**Software:** Shupeng Zou.

**Supervision:** Minghui Sun.

**Writing – original draft:** Qian Cheng.

## Supplementary Material


















