# Supplementary material for: Effect of ertugliflozin on renal function and cardiovascular outcomes in patients with type 2 diabetes mellitus: A systematic review and meta-analysis
Source: Medicine (Baltimore). 2023 Mar 10;102(10):e33198. doi: 10.1097/MD.0000000000033198 (PMC9997778; doi:10.1097/MD.0000000000033198)

Supplementary Material S6

Supplementary Figure S6 | Subgroup analysis of the effect of ertugliflozin on the eGFR of the T2DM participants based on eGFR of baseline.

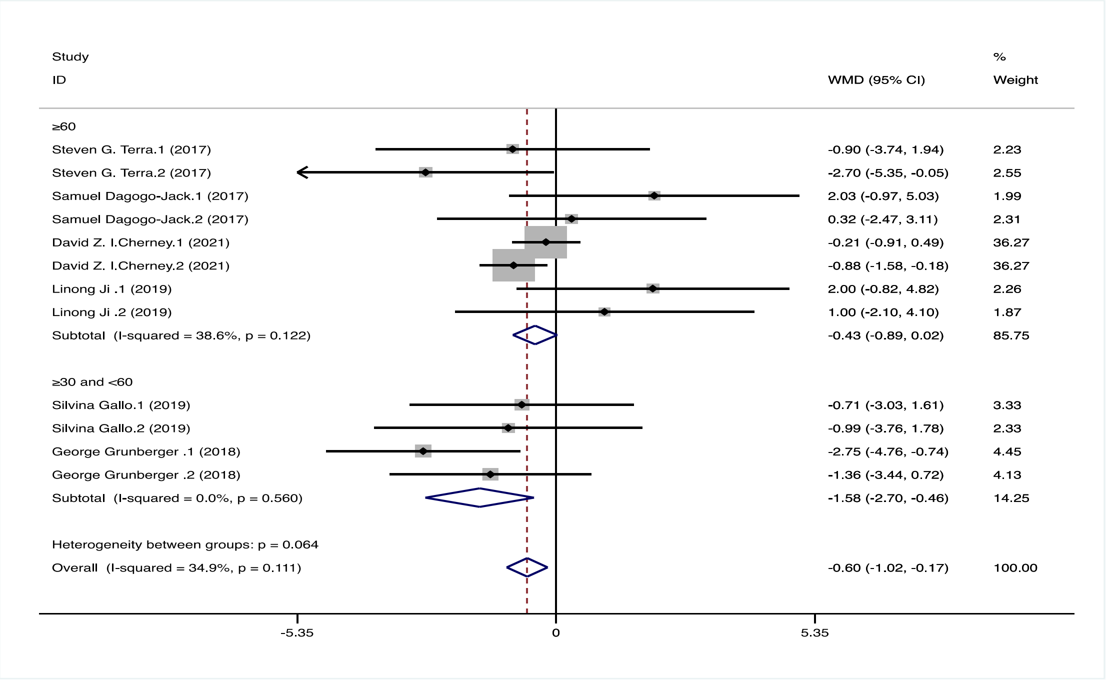

Supplement: Supplementary file 6 [file medi-102-e33198-s006.pdf]
