# Supplementary figures and images for: Effect of ertugliflozin on renal function and cardiovascular outcomes in patients with type 2 diabetes mellitus: A systematic review and meta-analysis
Source: Medicine (Baltimore). 2023 Mar 10;102(10):e33198. doi: 10.1097/MD.0000000000033198 (PMC9997778; doi:10.1097/MD.0000000000033198)

# Supplementary Material S9

Supplementary Figure S9 | Egger's test for publication bias ( $p=0.843$ ).

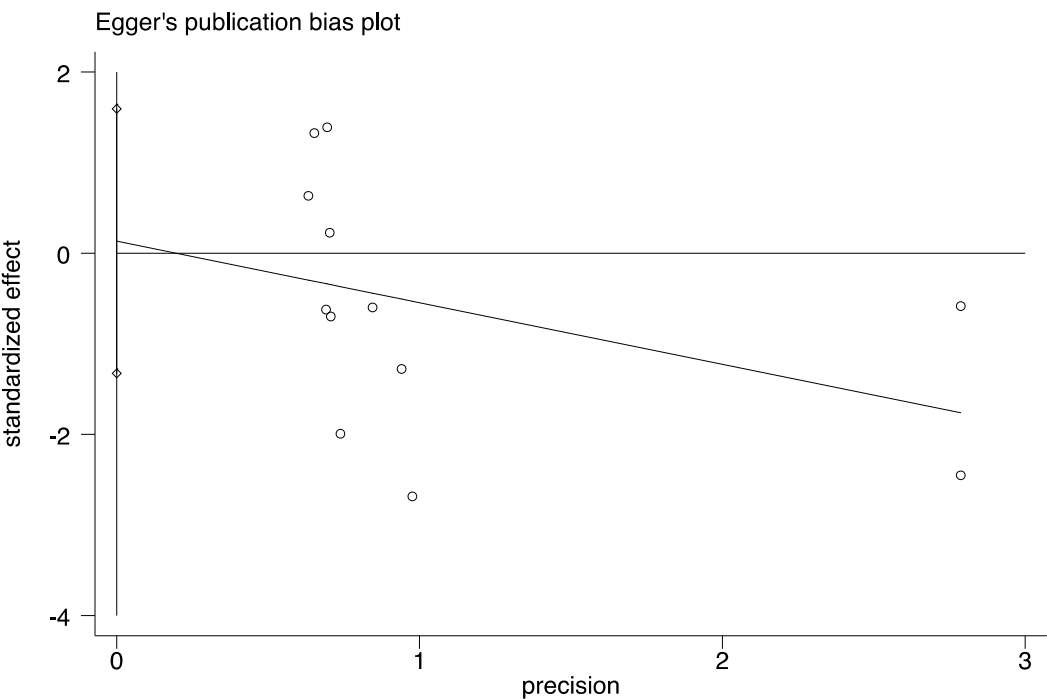

Supplement: Supplementary file 9 [file medi-102-e33198-s009.pdf]
